# The greater tuberosity version angle: a novel method of acquiring humeral alignment during intramedullary nailing

**DOI:** 10.1302/2633-1462.510.BJO-2024-0105

**Published:** 2024-10-22

**Authors:** Jose M. Gutierrez-Naranjo, Luis M. Salazar, Vaibhav A. Kanawade, Emam E. Abdel Fatah, Mohamed Mahfouz, Nicholas W. Brady, Anil K. Dutta

**Affiliations:** 1 Department of Orthopaedics, UT Health San Antonio, San Antonio, Texas, USA; 2 Department of Mechanical, Aerospace, and Biomedical Engineering, University of Tennessee, Knoxville, Tennessee, USA; 3 Department of Orthopaedics, University of New Mexico, Albuquerque, New Mexico, USA

**Keywords:** Humerus, Malrotation, Alignment, Greater tuberosity, Epicondylar axis, intramedullary nailing, humerus, transepicondylar axis, 3D CT scans, CT scanned, variance, intramedullary nail, Biceps groove, humeral fractures, imaging studies

## Abstract

**Aims:**

This study aims to describe a new method that may be used as a supplement to evaluate humeral rotational alignment during intramedullary nail (IMN) insertion using the profile of the perpendicular peak of the greater tuberosity and its relation to the transepicondylar axis. We called this angle the greater tuberosity version angle (GTVA).

**Methods:**

This study analyzed 506 cadaveric humeri of adult patients. All humeri were CT scanned using 0.625 × 0.625 × 0.625 mm cubic voxels. The images acquired were used to generate 3D surface models of the humerus. Next, 3D landmarks were automatically calculated on each 3D bone using custom-written C++ software. The anatomical landmarks analyzed were the transepicondylar axis, the humerus anatomical axis, and the peak of the perpendicular axis of the greater tuberosity. Lastly, the angle between the transepicondylar axis and the greater tuberosity axis was calculated and defined as the GTVA.

**Results:**

The value of GTVA was 20.9° (SD 4.7°) (95% CI 20.47° to 21.3°). Results of analysis of variance revealed that females had a statistically significant larger angle of 21.95° (SD 4.49°) compared to males, which were found to be 20.49° (SD 4.8°) (p = 0.001).

**Conclusion:**

This study identified a consistent relationship between palpable anatomical landmarks, enhancing IMN accuracy by utilizing 3D CT scans and replicating a 20.9° angle from the greater tuberosity to the transepicondylar axis. Using this angle as a secondary reference may help mitigate the complications associated with malrotation of the humerus following IMN. However, future trials are needed for clinical validation.

Cite this article: *Bone Jt Open* 2024;5(10):929–936.

## Introduction

Mid-shaft humeral fractures account for approximately 1% to 3% of all fractures.^1-4^ Conservative management with splinting, casting, and functional bracing remains the most popular initial treatment option, as the excellent blood supply from the muscles surrounding the humeral diaphysis provides a favourable healing environment.^4-6^ However, surgical treatment options, such as intramedullary nailing (IMN), open reduction and internal plate fixation (ORIF), minimally invasive plate osteosynthesis (MIPO), and external fixation, have all shown promising outcomes, although the ideal option remains controversial.^7-11^

Nevertheless, IMN is gaining traction in specific settings, such as complex segmental fractures, gunshot wounds, soft-tissue degloving injuries, and in the elderly population, as it is a less invasive procedure and has also displayed a significantly shorter operating time and time to union.^8-12^ When considering IMN for humeral fractures, rotational alignment of the humerus is challenging. Malrotation of the humerus during IMN could lead to decreased shoulder range of motion (ROM), increased shoulder dislocations, nonunion, and malunion.^13,14^ To the authors’ knowledge, there is little to no literature to guide surgeons through the nailing of the humerus to achieve proper alignment.

In previous studies, the bicipital groove has been reported as an anatomical reference point in shoulder arthroplasty to help attain appropriate humeral retroversion. As such, it has been considered a suitable landmark during IMN of the humerus.^15,16^ However, its helicoidal shape and groove orientation vary at the humeral anatomical neck and surgical neck, making it difficult for the surgeon to establish a superior approach during IMN, and may be susceptible to distortion by trauma.^17^ Boothby et al^18^ describe a fluoroscopic technique that utilizes the sulcus between the lateral head and greater tuberosity of the proximal humerus as a reference for rotational alignment, rather than relying solely on the bicipital groove.

A notable alternative, which the authors have long observed, is the conspicuous visibility of the apex of the greater tuberosity. This anatomical feature can be correlated with the transepicondylar axis, much like in the context of shoulder arthroplasty. Consequently, this study aims to address this limitation by introducing a novel measurement angle, the greater tuberosity version angle (GTVA), capable of identifying anatomical landmarks within a 3D space. The GTVA represents the geometrical relationship between the perpendicular axis of the greater tuberosity and the transepicondylar axis. It holds the potential to facilitate the alignment of the humeral shaft with its innate anatomical position during IMN for humeral shaft fractures.

## Methods

In this study, we analyzed the right and left cadaveric humerus of 506 individuals (138 females and 368 males). Those specimens are a subset of the William M. Bass donated skeletal collection at the University of Tennessee, USA. All humeri were CT-scanned using 0.625 × 0.625 × 0.625 mm cubic voxels. The DICOM images from acquired CT scans were then segmented, and surface models (meshes) were generated. This segmentation process has been proven reliable with a negligible interobserver error rate of 0.163 mm, intraobserver error of 0.105 mm, and pairwise interobserver variability of 0.269 mm.^19,20^

Segmented models for each humerus were added to the bone atlas. Briefly, a bone atlas is an average model that captures the primary shape variation of a bone and allows for the comparison of global shape differences between groups or populations, guaranteeing standardization, normalization, and landmark correspondence across a population. Additionally, it provides a mean for automated calculation of 3D landmarking.

**Fig. 1:**
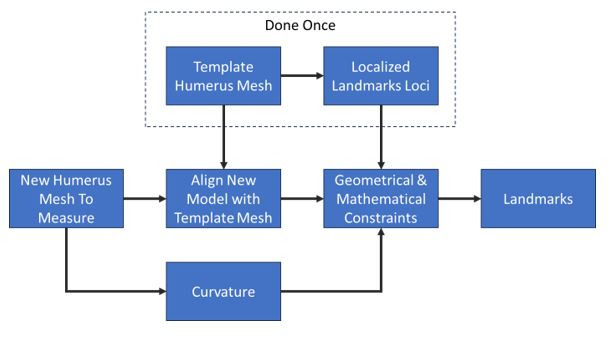
Flowchart outlining methodology of calculating automated measurements from the statistical atlas.

3D landmarks were automatically calculated on each 3D bone using custom written software in C++ (Institute for Advanced Materials & Manufacturing, USA), following a similar approach as defined by Mahfouz et al^19^ and Abdel Fatah et al.^20^ Landmarks sometimes falling between CT slices can be miscalculated in 2D analyses, which is particularly visible in the case of transepicondylar axis. Utilizing a 3D approach ensures all landmarks are anatomically accurate in three dimensions. Figure 1 outlines the algorithm used to calculate landmarks on the segmented humerus meshes automatically.

**Fig. 2:**
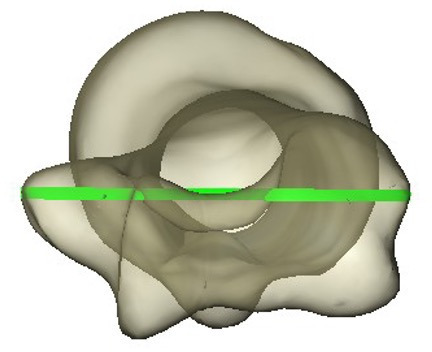
Transepicondylar axis of the humerus.

**Fig. 3:**
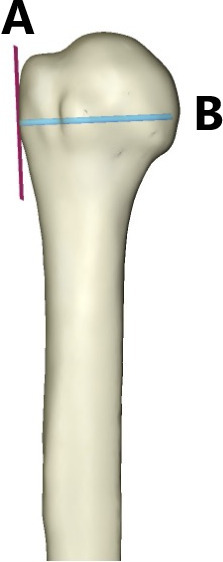
3D image of the greater tubercle of the humerus (A) and transepicondylar axis of the humerus (B).

The following landmarks were calculated: the transepicondylar axis (Figure 2) was defined as the axis connecting the two most prominent points on the medial and lateral epicondyles of the humerus. The greater tuberosity axis was then calculated by finding the most prominent point on the greater tuberosity, and its direction was approximated by computing the bone surface normal (a vector perpendicular to the bone surface) at that point (Figure 3).

**Fig. 4:**
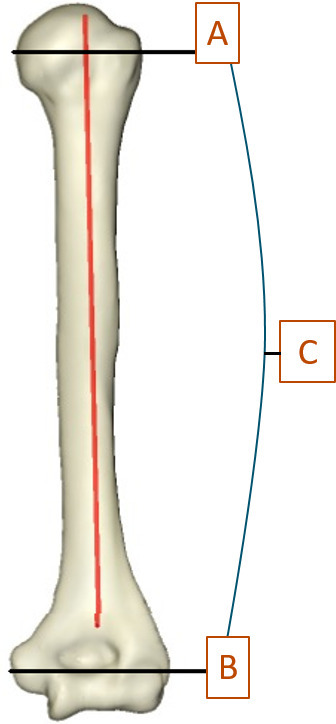
3D image showing the greater tuberosity axis (A) and transepicondylar axis (B), between which is the greater tuberosity version angle (C).

After establishing landmarks, the angle between the transepicondylar axis and the greater tuberosity axis was calculated and defined as the GTVA (Figure 4).

### Surgical technique

First, standard exposure of the proximal humerus is accomplished through a mini deltoid split approach. The nail entry position is typically at the essential apex, utilizing a rotator cuff split. A standard nail insertion is then performed for traditional IMN systems.

Next, the apex of the greater tuberosity is identified, and its perpendicular axis is marked, either with a small unicortical Kirschner wire, the direct lateral locking screw, or the targeting sleeve. This perpendicular point of the greater tuberosity serves as the first reference landmark or “peak” for the GTVA. Gross overall general alignment and fracture reduction are confirmed utilizing standard fluoroscopic evaluation techniques, as well as assessing the upper limb carrying angle. Once general alignment is satisfactory, fluoroscopic confirmation of nail reduction is performed.

**Fig. 5:**
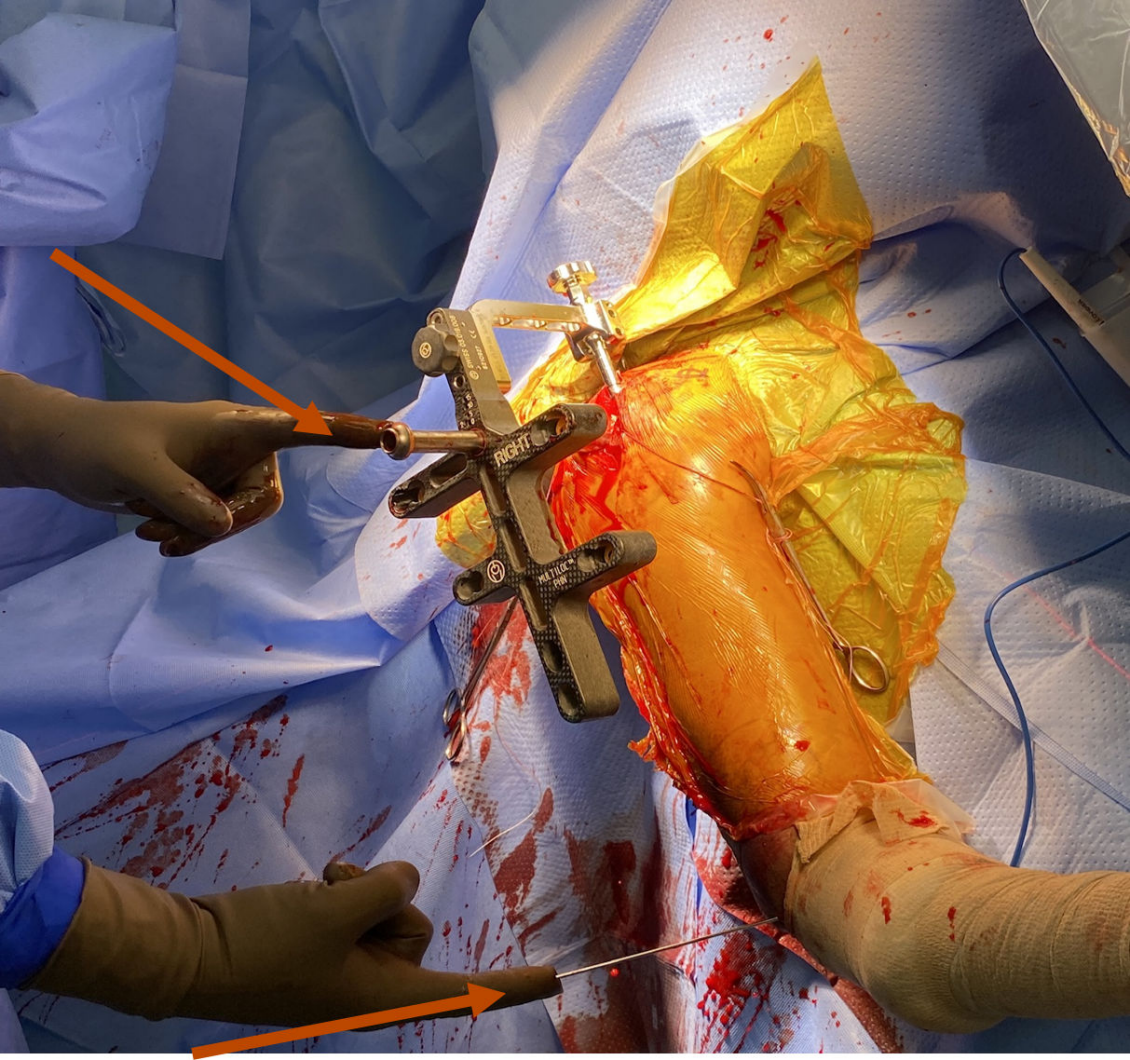
Anatomical landmarks or “peaks”. The greater tuberosity axis (top arrow). Epicondylar axis (lower arrow).

**Fig. 6:**
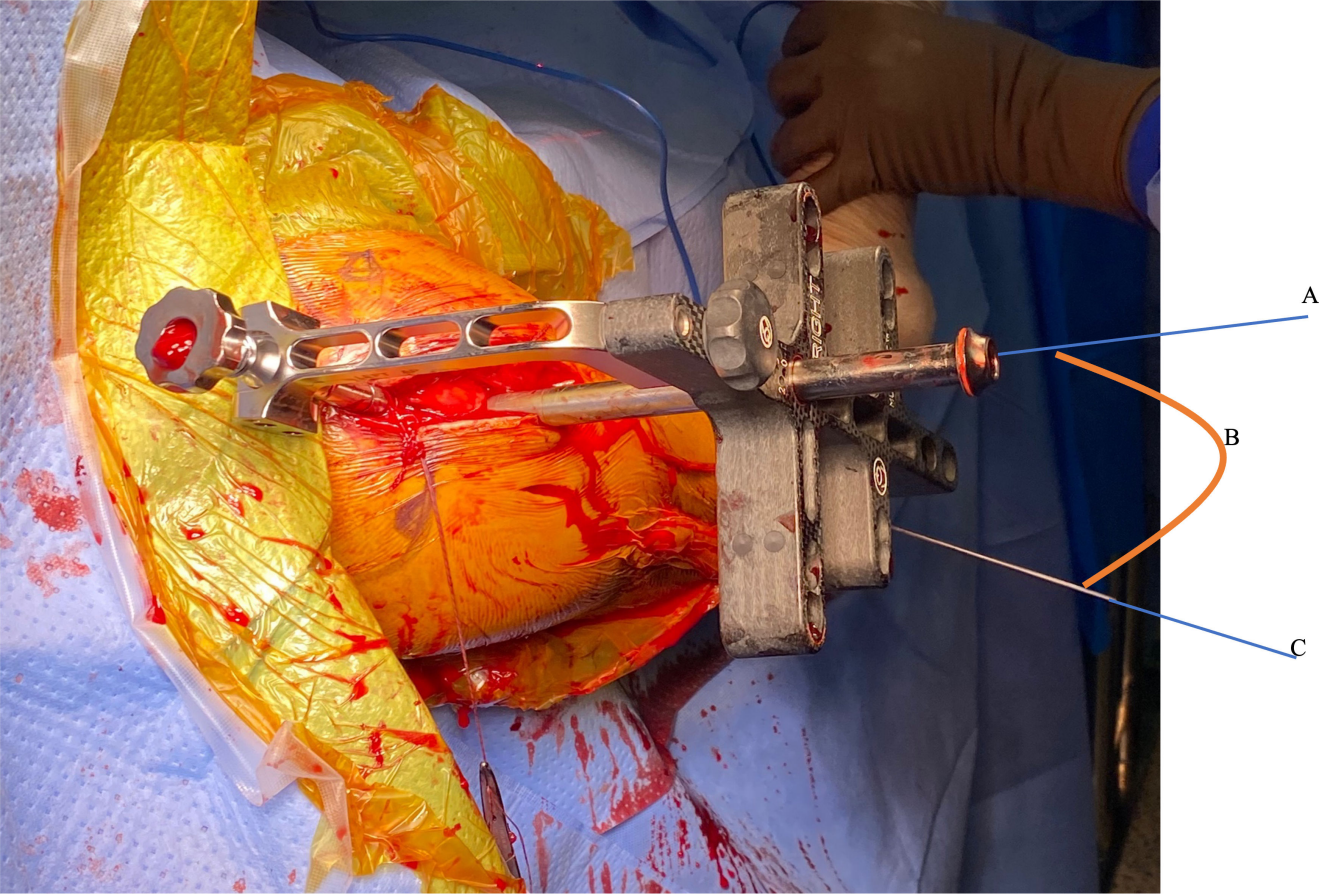
The greater tuberosity version angle technique (GTVA): the greater tuberosity axis (A); GTVA (B); and epicondylar axis (C).

An epicondylar guide is then used to insert a unicortical pin along the epicondylar axis, serving as the second reference point or “peak” (Figure 5). The rotational alignment is verified to be correct by direct visualization or by utilizing a goniometer to compare the angle between the epicondylar axis pin and the proximal locking screw or sleeve, setting it to the defined 20° angle relationship (Figure 6).

### Statistical analysis

Descriptive statistics of the greater tuberosity angle were analyzed using JMP Pro statistical software v. 15.2.0 (JMP Pro, USA). One-way analysis of variance (ANOVA) was used to examine differences in sex between males and females. Statistical significance was set at p < 0.05 for all analyses.

### Ethics approval

The Institutional Review Board (IRB) at UT Health San Antonio, USA, confirmed that no ethical approval is required.

## Results

**Fig. 7:**
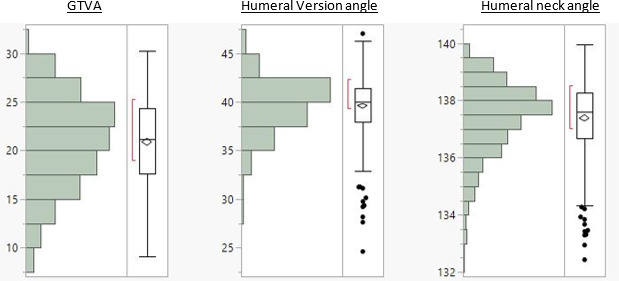
Data summary of the greater tuberosity to epicondylar axis, humeral version, and humeral neck angles (in degrees). Entire box = IQR; top box border = 75th percentile; bottom box border = 25th percentile; line inside box = median; X = mean; whiskers = 1.5 x IQR; and circles = outliers beyond 1.5.

The interobserver reliability tests yielded a negligible interobserver error rate of 0.163 mm, intraobserver error of 0.105 mm, and pairwise interobserver variability of 0.269 mm. The value of the GTVA was 20.9° (SD 4.7°) (95% CI 20.47° to 21.3°); further descriptive statistics and demographics are listed in Table I, Table II, and Figure 7. Results of ANOVA (Table III) revealed that females had a statistically significant larger angle of 21.95° (SD 4.49°) compared to males, which was found to be 20.49° (SD 4.8°) (p = 0.001).

**Table I.** Descriptive statistics.

Variable	GTVA, °	Humeral version angle, °	Humeral neck angle, °
Mean (SD)	20.88 (4.73)	39.58 (2.89)	137.39 (1.28)
25th/50th/75th percentile	17.6/21.2/24.36	37.93/39.99/41.39	136.68/137.61/138.26
Minimum to maximum	9.11 to 30.23	24.61 to 47.02	132.43 to 139.66

GTVA, greater tuberosity version angle.

**Table II.** Demographic data.

**Variable**	**Data**
**Sex, n (%)**	
Male	367 (72.5)
Female	139 (27.5)
**Race, n (%)**	
White	465 (92)
Black	41 (8)
Mean age, yrs (SD)	59.8 (19.6)

**Table III.** GTVA results by sex.

Sex	Number	GTVA, ° (SD)
Male	368	20.49 (4.80)
Female	138	21.95 (4.49)

p = 0.001.

GTVA, greater tuberosity version angle.

## Discussion

This study aimed to demonstrate a new clinically applicable technique to provide rotational accuracy during humeral IMN. To verify the relationship between the key anatomical landmarks involved, a large 3D cadaveric modelling system was employed, as these relationships are difficult to measure with precision in ingrowth specimen models.

Through 3D CT scans on a sizeable cohort, this study determined the precise relationship between the greater tuberosity’s perpendicular axis and the transepicondylar axis to be 20.9° (SD 4.7°). There is a lack of well-documented procedures in the literature describing verified ways to correct malrotation during IMN. To our knowledge, this study is novel in its introduction of a technique that establishes reliable and easily identifiable anatomical landmarks to achieve satisfactory humeral rotational alignment.

The GTVA has the distinct advantage of utilizing the greater tuberosity as a reference point, which is easily identifiable on imaging studies and clinically palpable intraoperatively. Our findings introduce a new method to mitigate complications associated with humeral IMN due to the sequelae of malrotation. Li et al^14^ found that up to 27.2% of patients undergoing IMN could experience malrotation. Moreover, they reported that the extent of malrotation was correlated with a limited shoulder ROM. Lin and Hou^21^ described similar findings in their study on rotational alignment after humeral closed, locked nailing. They also excluded patients with a nonunion, shoulder injury, pre-existing shoulder disease, and lack of participation in rehabilitation exercises, as these conditions produce a susceptibility towards a decreased ROM. Nevertheless, excessive malrotation was still reported as a significant cause of rotational limitation of the shoulder following surgery.

Previous studies have focused on various techniques to restore proper humeral alignment during IMN, such as patient and arm positioning to account for the carrying angle, intraoperative fluoroscopic assessment, and the use of the bicipital groove in relation to the transepicondylar axis as an anatomical landmark.^21-23^

While intraoperative fluoroscopic techniques for assessing rotational alignment have been described in the lower limb literature, they can be challenging to perform and may lack precision and reproducibility.^24,25^ The humeral anatomy presents unique difficulties in reliably assessing rotational alignment using standard fluoroscopic views alone. Intraoperative fluoroscopy is also routinely used to obtain adequate placement of prostheses radiologically, though it is challenging to acquire humeral alignment accurately through this method, particularly in comminuted and segmental fractures.

Unlike plate fixation, surgeons cannot directly visualize reduction with IMN, so using best-fit estimates through intraoperative radiographs can lead to an imprecise humeral position. Rommens et al^6^ studied 190 patients undergoing humeral IMN and concluded that a degree of malrotation of less than 20° leads to acceptable functional and cosmetic outcomes. This finding has been corroborated by multiple studies.^14,21,26^ As we can imagine, without a preoperative assessment of humeral alignment using 3D CT, it is not easy to estimate less than 20° of rotation using intraoperative fluoroscopy.

While other methods and anatomical landmarks have been proposed to confirm humeral alignment, such as utilizing the angle between the bicipital groove axis and the transepicondylar axis,^15,16,27^ these studies have notable limitations: the sample sizes were relatively small, and the measurement techniques employed may lack the desired accuracy.

Furthermore, the bicipital groove’s highly variable anatomy has led to hesitation to establish it as an anatomical landmark for reproducing humeral retroversion during shoulder arthroplasty.^28-30^ The literature shows that the bicipital groove orientation significantly differs in the surgical and anatomical necks of the humerus.^17,28,30^ Therefore, using the bicipital groove during IMN could introduce an error while attempting to recreate humeral alignment.

In a related context, surgical navigation systems could assist in identifying appropriate anatomical landmarks and enhance humeral alignment efficacy. In addition, these systems provide surgeons with improved visualization of target areas, precise tracking of surgical instruments, and more efficient execution of their approach.^31-33^ By integrating the recognized relationship between glenohumeral version angle and 3D CT scans with augmented visualization and guidance offered by navigation systems, surgeons may achieve precise alignment during IMN, potentially decreasing malrotation risk and its correlated complications. However, it is necessary to conduct further research to ascertain the feasibility and effectiveness of incorporating surgical navigation systems into this strategy. The benefits should be critically assessed, considering the complexities associated with implementing advanced healthcare technology.

We hypothesize that the GTVA technique will decrease the incidence of humeral malrotation and reduce the complications associated with IMN by predictably restoring humeral alignment. Another significant advantage of the GTVA is the ease of access during surgery and its reproducibility throughout the IMN process. Furthermore, less exposure is required to visualize the bicipital groove, thereby reducing the risk of further injury. Additionally, the direct lateral screw for most IM nails correlates with the apex of the greater tuberosity, facilitating the approach for the surgeon.

With these two reference points established, the nail is locked proximally and distally, utilizing the GTVA to provide additional rotational control and ensure proper humeral alignment.

The GTVA technique offers practical utility, especially in complex humeral fracture patterns such as comminuted or segmental injuries, where controlling rotational alignment can be challenging. The GTVA provides a valuable reference point by utilizing the easily identifiable greater tuberosity as an anatomical landmark. By correlating the apex of the greater tuberosity with the trajectory of the direct lateral interlocking screw, optimal nail positioning can be enhanced, both proximally and distally, for the majority of humeral nailing systems.

Nevertheless, our study has some limitations that must be addressed. First, the limits of variability remain unclear, and the role of comparison to the contralateral side was not evaluated. Second, this study was limited to a GTVA apex model based on cadavers, and intraoperatively, it may not be as accurate or easy to perform. Lastly, no clinical evidence was provided to demonstrate a difference in outcomes; therefore, more studies evaluating the GTVA technique are required to fully evaluate the potential benefits.

In conclusion, determining appropriate humeral nail insertion has been difficult due to the lack of anatomical landmarks and procedures in the literature. Inadequate nail insertion can lead to rotational deformities of the humerus, which has noteworthy associated complications. Our study has successfully identified a consistent relationship between anatomical landmarks that can significantly aid in determining appropriate humeral nail insertion. By utilizing 3D CT scans and replicating a 20.9° angle from the greater tuberosity to the transepicondylar axis, we can enhance the accuracy of IMN and minimize adverse outcomes. However, future prospective trials are essential to validate the effectiveness of our technique clinically.

**Take home message**

- An ongoing concern in intramedullary nailing (IMN) procedures has been the need for clearly defined anatomical landmarks to ensure proper alignment.

- This study presents the greater tuberosity version angle (GTVA), which provides a reliable reference point to mitigate the risk of malrotation-related complications during IMN procedures.

- Unlike the femur and tibia, where methods for evaluating rotational alignment exist, achieving standardized rotational alignment during humeral IMN has historically lacked a well-defined technique, relying primarily on overall limb alignment assessments.

- This study highlights the distinct advantages of utilizing the greater tuberosity as a consistent reference point.

- The greater tuberosity is an easily identifiable and visible anatomical landmark, both intraoperatively and on imaging studies, providing a practical and reliable guide for achieving proper humeral rotational alignment during nailing procedures. By using the greater tuberosity as a reference, surgeons can employ a standardized technique to optimize rotational alignment and reduce the risk of malreduction complications.

## Data Availability

The data that support the findings for this study are available to other researchers from the corresponding author upon reasonable request.
